# Treat staff like they make a difference, and they will

**DOI:** 10.1016/j.clinme.2024.100018

**Published:** 2024-01-24

**Authors:** Anton Emmanuel

It is an aphorism of business that the way in which staff treat customers is a reflection of how the organisation treats the staff. Regrettably, as health services the world face a number of crises, it is clear that the experience of their staff is also suffering. Some of those challenges, such as increasing complexity and workload volume, have been the subject of papers in recent editions of *Clinical Medicine*. A number of articles in this edition focus on the experience of the workforce as an additional dimension. Page *et al*^1^ describe the challenges faced by specialty and specialist (SAS) doctors. This group of colleagues in the UK NHS represent the fastest growing group of doctors and are often from minoritised backgrounds. They constitute a cohort who are frequently denied parity of opportunity to progress and fulfil career aspirations. This has been recognised for a long time and there is a welter of policy papers proposing the service corrections needed to appropriately develop these doctors – but, as is often seen at times of pressure in the system, it is those who are most in need of support who are least likely receive it. This qualitative paper goes beyond just detailing the challenges faced by SAS doctors: it also proposes the cultural and process changes that need to be addressed to create a level playing field. Another sphere of medicine where an adverse culture has been described is among academic clinicians, and this is often expressed in the processes that define academic progression, notably the Research Excellence Framework (www.ref.ac.uk). Davies *et al*^2^ describe how structures like the REF can not only perpetuate inequalities experienced by staff (especially women and ethnic minority researchers) but also influence the kid of research undertaken. There are also opinion pieces about the stigma around mental health issues experienced by doctors,^3^ and about a proposal to make the work and focus of research ethics committees more inclusive of the community of patients served.^4^

Respiratory medicine is also strongly in the spotlight in this edition. The CME section comprises five strong reviews on pulmonary infections.^5^, ^6^, ^7^, ^8^, ^9^ There is also a wonderful case-based manuscript^10^ on the management of the hypercapnic patient, covering both acute and clinic presentations. This paper exemplifies what Clinical Medicine prides itself on publishing – highly practical manuscripts, whether reviews or original research, that directly improve the confidence of practising physicians.
